# Formaldehydes in Feed and Their Potential Interaction With the Poultry Gastrointestinal Tract Microbial Community–A Review

**DOI:** 10.3389/fvets.2019.00188

**Published:** 2019-06-13

**Authors:** Steven C. Ricke, Kurt Richardson, Dana K. Dittoe

**Affiliations:** ^1^Department of Food Science, Center of Food Safety, University of Arkansas, Fayetteville, AR, United States; ^2^Anitox Corporation, Lawrence, GA, United States

**Keywords:** formaldehyde, feed, poultry, *Salmonella*, gastrointestinal tract

## Abstract

As antibiotics continue to be phased out of livestock production, alternative feed amendments have received increased interest not only from a research standpoint but for commercial application. Most of the emphasis to date has focused on food safety aspects, particularly on lowering the incidence of foodborne pathogens in livestock. Several candidates are currently either being examined or are already being implemented in commercial settings. Among these candidates are chemical compounds such as formaldehyde. Formaldehyde has historically been used to inhibit *Salmonella* in feeds during feed processing. Currently, there are several commercial products available for this purpose. This review will cover both the historical background, current research, and prospects for further research on the poultry gastrointestinal tract and feeds treated with formaldehyde.

## Introduction

Treatment of animal feeds has always been considered a critical component to food animal management to prevent the formation of mycotoxins and other biological contributors to contamination and feed quality decline during storage (1). There have been numerous research studies and applications for various chemicals to be added to animal and poultry feeds during feed processing, and these have been well-documented in published review articles over the past few decades (1–5). Not surprisingly much of the focus for the application of chemical additives particularly in poultry feeds has been directed toward limiting *Salmonella* in the feed (2–7). Research studies have ranged from the assessment of feed contamination at the feed mill to bird feeding trials and involved both natural *Salmonella* contamination and inoculation of a marker *Salmonella* strain.

In order for a particular chemical feed additive to possess commercial attractiveness to be promoted for routine use in animal and poultry feeds, several criteria essential to meet this demand would have to be considered. Some of these specifically for feed antimicrobials have been outlined previously (3) but would still apply in a general sense. Effectiveness in the presence of a high organic load that is characteristic of a typical mixed feed and/or individual feed ingredient would be a must. The effective dose would have to be safe in the target animal and not result in undesired residues in animal products. The relative cost to be applied to large bulk quantities of feed would also need to be of a commercial scale level of utility as well as ease of application and minimal damage to milling equipment. Governmental regulatory approval both domestically in the United States and internationally for use in animal diets should be in place. The worker safety during application in the feed mill and post-milling, delivery to the farm and use at the farm would have to be established.

In this review a discussion of *Salmonella* occurrence in feeds will be described in brief, followed by discussion of one of the more prominent and widespread used chemical group of compounds, namely, aldehydes with the primary focus on formaldehyde/formalin in terms of antimicrobial mechanism(s) and efficacy as feed additives in the poultry gastrointestinal tract. Finally, future directions for application and improving efficacy will be discussed.

## Formaldehyde—Natural Occurrence and Biological Applications

In general aldehydes are relatively ubiquitous in the environment originating not only as a natural compound but as an intermediary endogenous product in biological metabolism and other processes as well-generation from automobile exhaust gases and indoor environments from sources such as building materials and furniture (8–10). The chemistry and pathways for their formation have been extensively discussed in a review by O'Brien et al. (8) and will not be discussed in detail in the current review. Numerous aldehydes including formaldehyde are detectable in a wide range of foods including fruits, vegetables, meat, cheese, and seafood (8, 11, 12). Aldehydes can be detected in the air, feed, tissue, and feces via personal monitors, spectrophotometric measurement of color reaction between tissue distillate, and chromatographic-sulphuric acid reaction, respectively (13). They can be formed as volatile aldehydes during cooking, particularly from edible oils, auto oxidation of unsaturated fatty acids, odor compounds emanating from rancid high-fat foods, and occurring as products from the storage of beer (12). Aldehydes and ketones are known to increase during milk thermal processing and storage of milk powder, resulting in changes in flavor and milk powder porosity (14). Formaldehyde in foods is released in the stomach and absorbed into the bloodstream where it is metabolized to formic acid by the red blood cells. Formic acid is further metabolized to carbon dioxide and water (15, 16). The metabolic half-life of formaldehyde is 60 to 90 s. This route of metabolism may be similar for other aldehydes. Aldehydes are also an important set of useful compounds for industrial processes such as flavors, fragrances, and pharmaceutical precursors. In addition, efforts have been made to genetically modify microorganisms to accumulate sufficient quantities for commercial purposes (17, 18).

Formaldehyde can serve as a fixative preventing cell autolysis and reacting with proteins, lipids and nucleic acids (19–21). The interaction of formaldehyde with peptides has been characterized by Metz et al. (17) as occurring via formation of either methylol groups, Schiff bases, or methylene bridges. Methylol and Schiff base modifications are considered reversible whereas methylene bridge products are stable and can lead to cross-linking of protein chains (17, 22–24). The type of bond formed between formaldehyde and protein/ amino acids is dependent on the reaction conditions (25).

The reaction of formaldehyde with aqueous solutions of crystalline amino acids (98:2 ratio) at 24°C resulted in the formation of a compound (described as an adduct) exhibited antimicrobial activity against *E. coli* and *Salmonella* (26). Only lysine, arginine, histidine and asparagine were reported to form the adduct. The bond between lysine and formaldehyde was found to be reversible and was broken by distillation in a mildly acidic solution suggesting a methylol or Schiff base linkage. This is consistent with the findings of Alexander et al. (25) that reported that methylol derivatives of formaldehyde and amino groups are unstable and dissociate under mildly acidic conditions. Additional research by Barlow (27) and Rude et al. (28) indicate formaldehyde added to fishmeal or corn amended with crystalline lysine under mild reaction conditions (ambient temperature) does not affect availability at the 3 kg/ton level.

The ability of formaldehyde to form methylene bridges and cross-link protein was first utilized to improve the elasticity of wool. Intensive research has been conducted in this area and various reaction conditions utilized. Reaction conditions required to cross-link amino have been found to be dependent on the ratio of formaldehyde to protein, reaction temperature, reaction time and pH (25). Theis and Jacoby (29) first reported that protein could be cross-linked by formaldehyde when a 3:2 ratio of amino acid to formaldehyde was incubated at 60°C for 30 min, but that at a 3:1 ratio of amino acid, the bond was reversible. Other researchers have utilized higher reaction temperatures (up to 100°C), longer reaction times (up to 24 h) and higher formaldehyde to protein ratios to form a cross-linked protein (25, 30). However, the interaction of formaldehyde with proteins may be somewhat more complex and variable compared to isolated peptides. For example, formaldehyde peptide cross-linking has been examined in more detail by Toews et al. (31) who reported that some regions within proteins are more susceptible to formaldehyde cross-linking than other regions of the respective proteins, and the variation in three dimensional structures of proteins dictate relative reactivities to formaldehyde (31).

Regardless of the exact mechanism(s) in which proteins are cross-linked, exposure of proteins to formaldehyde results in decreased water sensitivity, and increased resistance to chemical and enzyme exposure (22). This has been used for several practical applications in biology. Historically, formaldehyde has been used as a tissue fixative for clinical sample preservation that ensures stability for several years (32, 33). The ability to modify proteins has been taken advantage in the process of inactivating bacterial toxins and viruses for generating vaccines (17). Formaldehyde has also been implemented as a means to stabilize and retain intact whole cells, particularly bacteria. This has been used to preserve a consistent set of rumen bacterial cells to serve as an agent for immunization in layer hens to generate egg yolk polyclonal antibodies (34). Fixation of bacterial cells harvested after growth in large scale growth vessels and subsequent addition of thimerosol allowed for extended frozen storage of whole intact cells without the growth of bacterial contaminants until they could be used to immunize hens (35).

Using formaldehyde to stabilize bacterial cells has benefits for other types of studies where retaining intact whole cells may be critical. For example, formalin solutions have also been used to harvest and preserve rumen bacterial cells after continuous culture growth studies for cell dry weight determinations (36–38). Isaacson et al. (36) incorporated formalin fixation as part of the recovery process due to concerns over cell lysis occurring during the centrifugation and washing steps of mixed cultures that could impact the accuracy of dry weight estimates of rumen bacterial populations from continuous cultures. They concluded that the addition of formalin did not alter the dry weight results appreciably to impact the interpretation of the dry cell data. In a more recent study, Baker et al. (39) used formalin solutions to preserve pathogenic *Escherichia coli* strains for use in flow cytometry detection. In their study, there was a need to standardize an immuno-based flow cytometry analyses with known quantities of particular pathogenic *E. coli* pure culture isolates to serve as standards before assessing food samples. In this particular study, they demonstrated that formalin preserved sets of *E. coli* could be spiked into ground beef samples, recovered, quantified by both quantitative polymerase chain reaction and flow cytometry, and demonstrated that the two methods did not statistically differ from each other. They concluded that formalin fixed solutions of pathogenic *E. coli* could serve as internal standards for calibrating flow cytometry-based assays by providing stable known quantities of *E. coli* cells.

## Formaldehyde—Poultry Applications and Antibacterial Mechanism(s)

Given the ability of formaldehyde to interact with macromolecules and serve as a fixative agent for bacterial cells it is not surprising the formaldehyde would be a potential antimicrobial compound. Glutaraldehyde-based chemicals have been used for sterilization in clinical settings such as dental, medical and veterinary surgical facilities (40). Glutaraldehydes have also been employed as disinfectant sprays in broiler and animal housing and livestock transportation vehicles for limiting bacterial and viral contamination (41–45). Formaldehyde fumigation has been used for eggshell surface decontamination, but hazard concerns have motivated research for alternative methods that are as effective as formaldehyde in reducing bacterial loads even though formaldehyde remains one of the more effective antibacterials that are available (46–52). While it has been noted by Carrique-Mas et al. (53) that there are concerns regarding the safety of formaldehyde to humans, in order to reduce occupational exposure, formaldehyde is applied in an enclosed system [mixer/enclosed auger; USDA (54)]. In a recent risk assessment, the European Food Safety Authority indicated that formaldehyde would not be considered a risk to humans when employed as an animal nutrition product, but anyone handling the product should avoid exposure to the respiratory tract, skin, and eyes (3, 55, 56).

Historically only limited microbial data responses mostly based on culture methods have been generated for evaluating aldehyde disinfectants in poultry operations (46–52). Consequently, microbial profiling is confined to which media is used, the respective selective processes, and the segment of the microbial population capable of forming visible colonies. Now that microbiome sequencing has become routine, more comprehensive microbial community profiling has become possible to conduct a comparative assessment of disinfectant treatments on microbial populations such as those that inhabit poultry houses. For example, Jiang et al. (45) compared different disinfectant sprays and reported that glutaraldehyde not only reduced overall airborne bacterial contamination in empty broiler houses but based on 16S rDNA sequencing using an Illumina HiSeq sequencer, decreased the number of detectable phyla by nearly half (from 32 phyla to 17 phyla) compared to the non-disinfected house. Phyla diversity was even more substantially decreased (6 phyla detected) when a disinfectant mixture (aldehyde, alcohol, and quaternary ammonium salt) was used leading the authors to suggest a much broader antibacterial spectrum for the disinfection mixture. In future studies, it would be of interest to conduct metagenomic profiling to determine the frequency of antibacterial resistance genes in these microbial populations that are specific to certain disinfectants being implemented routinely.

**Figure 1:**
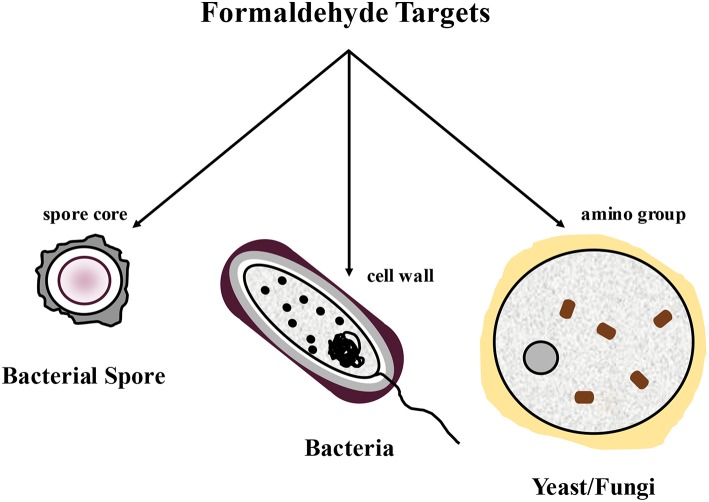
The impact of the use of formaldehyde on bacterial spores, bacteria, and yeast/fungus by targeting key components.

Formaldehyde was first utilized in the animal feed sector as a mold inhibitor for the preservation of high moisture corn (57). Formaldehyde has also been used extensively as a feed chemical antimicrobial to reduce *Salmonella* and improve general bacterial hygiene in feeds [Figure 1^1^, (3, 4, 53)]. In general, potential cell targets of formaldehyde include the spore cores of bacterial spores, the cell walls of bacteria, and the amino groups of fungi (58, 59). The antimicrobial activity of glutaraldehyde and formaldehyde is believed to be elicited primarily by both the formation of a Schiff base product and irreversible cross-linking of proteins, RNA, and DNA in bacteria and of proteins in feeds (3, 4, 26, 53, 58, 59).

Unlike some of the other feed additive acids that have been used over the years, little bacteriological work has been conducted to determine mechanisms of formaldehyde exposure on *Salmonella*. Temcharoen and Thilly (60) examined toxic and mutagenic effects of formaldehyde in a mutant *Salmonella* Typhimurium test strain that lacked either membrane translocation or phosphoribosyl-transferase. The basic concept in using the *Salmonella* tester strain (his^+^ revertant of an Ames *Salmonella* tester strain) is that if a particular compound is mutagenic then the histidine auxotrophic version of the tester strain will revert to a version that no longer requires histidine and can grow on media plates without histidine supplementation (61). Based on their results, Temcharoen and Thilly (60) concluded that formaldehyde was toxic and mutagenic to the *S*. Typhimurium tester strain and the minimum concentration required to induce mutagenicity was 0.167 mM. They hypothesized that formaldehyde may lead to mutations either by direct interaction with the bacterial cell's DNA, or reacting with amino groups, simple amines, amino acids, nucleic acids, or proteins to form mutagenic product(s).

As of date, there is no clear evidence linking the use of formaldehyde in poultry operations to the expression of resistance factors in *Salmonella*. For example, when *Salmonella* isolates exposed to different disinfectants including formaldehyde in Danish broiler houses were characterized by Gradel et al. (62) for minimum inhibitory concentrations (MIC), no clear-cut association could be detected among serovar persistences, tendencies to persist, or use of a particular disinfectant. Likewise, *S*. Enteritidis isolates from egg-laying flocks where a quaternary ammonium-formaldehyde disinfectant was used also did not exhibit alterations in susceptibility/resistance responses (63). This again proved to be true in *Salmonella* isolates known to be persistent in a fish feed plant (64) where even though these isolates had been exposed to a commercial organic acid- formaldehyde mixture they were no more resistant to disinfectants than *Salmonella* isolates from other sources. In feed applications as a chemical antimicrobial additive, formaldehyde is unlikely to directly interact with *Salmonella* cells in a fashion similar to the pure culture *Salmonella* incubations conducted by Temcharoen and Thilly (60) as described above. Instead, it is much more likely to chemically interact with the proteins present in feeds upon exposure and potentially affecting bird performance.

## Poultry Feeds and Microbial Contamination

Biological contamination of feeds by organisms has always been considered a complex issue with numerous factors influencing levels and types of organisms likely to be present on a particular feed or feed ingredient at any given time or location as previously discussed (1, 3, 65–67). Although few conclusions can be drawn, the microbial composition associated with animal and poultry feeds can be quite diverse (1, 3, 68). Prokaryotes, bacteriophage, fungi, and yeast have all been identified in feeds and in some cases isolated from a wide range of feeds (1–3, 69–72). Detecting particular patterns or critical factors that dictate specific bacterial and/or non-bacterial populations associated with feed remains elusive. Indeed, factors such as environmental conditions during storage and subsequent feeding to animals, storage time, and feed treatments would be expected to contribute to the final composition of a feed or feed ingredient but to what degree and what other factors may be involved remains unknown. As molecular techniques develop, it is conceivable that such methods could be employed to begin comprehensive studies that establish signature populations in the feed that do correlate with certain influential factors and potentially identify which factors are most critical to certain feed processing operations.

Among the bacterial contaminants potentially present in animal and poultry feeds several organisms would also be considered foodborne pathogens that could cause disease in humans. These include *Salmonella, Clostridium perfringens, Clostridium botulinum*, and *Listeria*, some of which have been more frequently identified with feed than others (2, 3, 73–77). Of the foodborne pathogens isolated from feeds, foodborne *Salmonella* serovars have received the most attention particularly with poultry feeds and feed ingredients and remain an issue for all aspects of vertically integrated poultry operations (6, 78–82). *Salmonella-*contaminated feed certainly has to be considered a potential risk factor for salmonellosis.

Poultry feed has been known to be a source of *Salmonella* since 1948 (83). In integrated operations, *Salmonella* control typically begins at the breeder level (84). Snoeyenbos (85) reported that the transmission of *Salmonella* in breeder eggs occurred with sufficient frequency to require control measures for *Salmonella* at the breeder and multiplier level. Wilding and Baxter-Jones (86) estimated that colonization of one breeder/multiplier by *Salmonella* might affect 65 broilers. Shapcott (87) reported that the presence of *Salmonella* in breeder feed might impact the transmission to broiler chicks. After implementing a rigorous program for the control of *Salmonella* at the breeder farm, the hatchery and the feed mill, both the broiler and breeder operations were *Salmonella* negative for >3 years. However, in June of 1980, a single breeder feed tested positive for *Salmonella* Sofia. Within 1 year, 100% of the flocks tested positive for *S*. Sofia. Jenson and Rosales (88) reported that 80% of the *Salmonella* serotypes found in breeder feed might be detected weeks later in breeder birds or their offspring.

The significance of *Salmonella* in feed and animal produce is less understood. There are many vectors for *Salmonella* transmission to poultry and animal produce, including breeders, hatchery, farm, feed mill, and the processing plant. Morris et al. (89) first discussed *Salmonella* in feed and its association with processing plant contamination. Of 12 serotypes of *Salmonella* isolated from the processing plant, six isolates were also present in feed. Only *S*. Montevideo isolates displayed a relationship in the frequency of detection between the feed mill and processing plant. Lahellec and Collins (90) reported that 8 of 16 serotypes of *Salmonella* isolated from the processing plant were found in feed. In a 3 year study of a large integrated broiler operation, McKenzie and Bains (91) observed that *Salmonella* in broiler carcasses displayed a 100% correlation with *Salmonella* in feed ingredients and grains. In Europe where *Salmonella* contamination rates of feed are low (<2%), Davies et al. (92) used a slightly different approach to determine if *Salmonella* in feed was associated with processing plant contamination. During a 2 year study, samples of dust and residues from feed mills of two large integrated broiler operations were analyzed for *Salmonella*. Corry et al. (93) compared isolated serotypes from feed to those present at the processing plant and found that 55% of *Salmonella* isolates from the processing plant originated from the feed. The connection of potential for salmonellosis to feed has been made in other ways as well. As an illustration of this particular point, Bucher et al. (94) characterized *Salmonella* isolates from chicken nuggets, strips, and pelleted broiler feed and concluded that *Salmonella* strains isolated from broiler feed were indistinguishable from isolates recovered from packaged raw, frozen chicken nuggets, and strips. Similar observations have been noted in commercial egg operations. Shirota et al. (95, 96) reported that both the frequency and the serotypes of *Salmonella* in feed were correlated to the frequency and serotypes of *Salmonella* in eggs (58% of egg isolates was identical to feed isolates). The authors of these studies concluded that *Salmonella* contamination of carcasses and egg contamination could be significantly reduced by minimizing the incidence of *Salmonella* in the feed. This would suggest that *Salmonella* possesses the capability of being transmitted from feed production, broiler growout/egg production, poultry processing and eventually retail establishments.

As a result of the widespread prevalence of *Salmonella* spp. in the environment and its capacity for survival under relatively harsh conditions such as increases in temperature (97–99) it is not surprising that *Salmonella* would come in contact with different stages of feed production all the way from cereal grain harvesting to feed milling and in turn lead to cross-contamination in places such as feed mills (3, 6, 78, 80, 81, 100–105). It is clear that better tracking methods will be needed to pinpoint ultimate origins for particular *Salmonella* spp., but this will be somewhat of a challenge given the high number of serovars that have been identified. Likewise, this makes developing effective control measures difficult due to the complexity of *Salmonella* occurrence in all phases of feed production and the range of potential *Salmonella* serovars that could be contaminants.

## Formaldehyde—Feed Studies

Given the effectiveness of formaldehyde as a general sanitizer, it is not surprising that there would be interest in applying it as an antimicrobial treatment for poultry feeds. Duncan and Adams (106) examined the use of formaldehyde gas as a potential treatment to fumigate feeds and eliminate *Salmonella* loads using chick starter, fish meal, and meat and bone meal artificially contaminated with *S*. Senftenberg as their test model. They initially tested a commercial acid-based blended product containing propionic acid, isopropyl alcohol, and phosphoric acid but found this to be relatively ineffective at reducing *S*. Senftenberg levels in the various feeds. Following this experiment, they formaldehyde fumigated contaminated feed samples at 37°C and 60% relative humidity in a forced-draft incubator. They concluded that 5 min of formaldehyde fumigation was adequate and that the maximum fumigant penetration was <2.54 cm, but at least 1.91 cm and effective depth was increased to over 5 cm for 500 gm samples if they were continuously mixed.

While formaldehyde fumigation applications were initially tested, formaldehyde liquid solutions that could be incorporated/mixed directly into the feed matrices were examined as potential chemical feed additives to feeds as a means to reduce *Salmonella* contamination. Moustafa et al. (107) artificially contaminated commercial poultry with *S*. Typhimurium after the feeds had initially been sterilized via autoclaving. They concluded that a 40 % formaldehyde solution applied at a rate of 10 L/ton resulted in complete reduction of *S*. Typhimurium within the first hour of treatment while only 94% reduction was achieved with a 5 L/ton rate during this same application time frame. More recently, Sbardella et al. (108) examined the effect of a 3.0 g per kg formaldehyde-propionic acid blend on natural bacterial populations in pig feed and reported reductions in natural populations of the enterobacteria populations. Based on these studies it appeared that formaldehyde solutions could be directly added to feeds and once mixed into the feed were effective in substantially reducing *Salmonella* contamination.

Studies on the residual activity of formaldehyde treated feed/ingredients to prevent recontamination by *Salmonella* was first reported by Barlow et al. (27). Fishmeal was treated with a formaldehyde-based product at 2 kg/ton and subsequently challenged with 200–500 cfu/g of *S*. Senftenberg and the time required to kill *Salmonella* determined. At 2 kg/ton, 5 to 9 days were required. When fishmeal was treated with 3 kg/ton and challenged with 1,500 to 2,000 cfu/g, all *Salmonella* was eliminated within the first 24 h. A similar study was conducted by Primm (109) using a mixed culture of *Salmonella* serotypes and higher challenge rates. At a challenge rate of 3,400 cfu/g, no *Salmonella* was detected at 3 kg/ton. The 3 kg/ton failed to protect the feed at challenge rates of >34,000 cfu/g.

## Formaldehyde Comparative Studies with Other Feed Additives

Commercially, there are several chemical options for treatment of feeds to control *Salmonella* as described in several reviews published over the years (1–7). From a management perspective it is important to be able to compare various sanitizers to identify either single compounds or combinations that are optimal for the particular conditions they are being applied. Along these lines, studies have been conducted over the years to directly compare feed additive organic acid blends with formaldehyde. In early work Smyser and Snoeyenbos (110) compared 12 different compounds as antimicrobials for *Salmonella* when these compounds were added to meat and bone meal (MBM). Several acids and non-acid antimicrobials were examined including among others, acetic acid, oleic acid, propionate salts, benzoic, sorbic, methylparaben, formalin at 0.05, 0.1, 0.12, and 0.2 % (w/w) and some commercial blends. A nalidixic acid resistant *S*. Infantis strain was used as the marker strain to inoculate the samples set at a moisture level in the MBM to support *Salmonella* growth. Plate enumerations were conducted beginning at 2 to 3 days post-inoculation and subsequently continued for anywhere from 1 to 2 weeks afterwards. All compounds except formalin at levels >0.1 % failed to prevent *S*. Infantis growth. The authors noted that while initial declines in *S*. Infantis occurred for many of the additives, the pH of the feed mixtures also became alkaline over time with spoilage ensuing.

Smyser and Snoeyenbos (110) commented that from their previous work that most of these compounds including formalin had minimal effect on *Salmonella* in MBM when added to the MBM matrix with a much lower moisture content. This would suggest that water activity is an important component for ensuring optimal antimicrobial activity. In a more recent study, Carrique-Mas et al. (53) used a spray application of a *Salmonella* inocula to a feed matrix to compare the respective efficacies of four different commercial organic acid (various combinations of formic, propionic, and sorbic acids) and formaldehyde-based feed additives in either fishmeal or MBM. The *Salmonella* inocula (*S*. Enteritidis, *S*. Typhimurium, *S*. Senftenberg, and *S*. Mbandaka) were sprayed onto the feed matrix accompanied by mixing, subsequently allowed to incubate over time followed by recovery for pre-enrichment. A critical outcome of the research results noted by the authors was that the carryover of the antimicrobials into the recovery media in turn appeared to “mask” and/or reduce the population recovery levels of the inoculated *Salmonella* and thus led to an overestimation of the antimicrobial effect due to decreased levels of *Salmonella* surviving in the recovery media. To counter this masking effect, the authors employed antimicrobial neutralizing antagonists such as histidine for formaldehyde or sodium hydroxide for organic acids to the pre-enrichment media to neutralize artifactual antimicrobial decreases resulting from the respective feed additive to add. One of the formaldehyde-based treatments elicited less masking and more efficacy against *Salmonella* with no differences among the serovars. Clearly, as more feed studies are done, caution will need to be exercised to avoid *Salmonella* methodology misinterpretations occurring from masking regardless of the antimicrobial used. This will mean that some quantitative methodology validation will need to be conducted to ensure that the results represent the *Salmonella* populations originally present in the feed matrix after treatment of the feeds. This may not only be a concern for *Salmonella* but may need to be considered for all non-*Salmonella* bacterial population enumerations to avoid artificial selection by masking in either the dilutions or the plating media.

Other factors for optimizing feed treatments to control *Salmonella* may be influential as well. Carrique-Mas et al. (53) pointed out that the timing of when a feed additive is applied could be important as they and others (111) have noted that pretreatment with organic acids and formaldehyde prior to inoculation of *Salmonella* results in a more rapid decline in bacterial populations suggesting that pretreated feeds may be more resistant to subsequent contamination. This has practical significance as the potential for *Salmonella* cross contamination during milling is considered a concern. This is illustrated in a study by Jones and Richardson (80) where they detected *Salmonella* recontamination originating from dust in the feed mill. This led them to conclude that potential cross contamination between areas of the mill operation are possible and must be taken into account as part of control strategy for *Salmonella* feed contamination. Even if *Salmonella* levels in feed are initially decreased during milling, risk of exposure to *Salmonella* remains. For example, Jones (81) concluded that thermal processes such as pelleting could reduce *Salmonella* levels, but recontamination could occur post-pelleting and suggested that the addition of chemical disinfectants could diminish potential recontamination.

There are strategies that can be utilized to limit recontamination. To this point, Cochrane et al. (112) examined post rendering chemical treatment of rendered feed ingredients by comparing a wide range of feed additives including a medium chain fatty acid (MCFA) blend (caproic, caprylic, and capric acids) with an organic acid blend (lactic, formic, propionic, and benzoic acids), an EO blend (garlic oleoresin, tumaric oleoresin, capsicum oleoresin, rosemary extract, and wild oregano), sodium bisulfate, and a commercial formaldehyde product. They initially treated the rendered protein feed ingredients (feather meal, blood meal, MBM, and poultry by-product meal) with the corresponding feed additive followed by spray inoculation with a *S*. Typhimurium strain. They observed that the feed ingredient matrix impacted *Salmonella* persistence as similar populations were recovered from both blood meal and MBM and, in turn, were higher than the populations enumerated from feather meal and poultry by-product meal. Out of all the products examined, they concluded that the MCFA blend and the formaldehyde commercial product were the most effective in preventing *S*. Typhimurium post processing contamination, but time and feed matrix type were all factors in reducing *S*. Typhimurium levels.

In summary, formaldehyde is an effective control agent for limiting *Salmonella* in feeds but when and where to apply it to achieve maximum efficacy needs to be standardized. This can be accomplished by developing a more complete picture of the microbial ecology of feed production (3). Understanding the microbial ecology of the feed mill as well as the feed ingredient and mixed feed matrices could potentially be helpful not only for *Salmonella* tracking but general microbial contamination that occurs in feed processing. While many of these non-*Salmonella* organisms are probably not deleterious to animals and/or humans their presence could be indicative of the impact of processing environmental conditions on the feed prior to being fed to the animal.

Application of next-generation sequencing (NGS) technologies would offer a more complete profile of the microbial population and depending on the bioinformatics analysis identify core feed microbial populations that align with certain characteristics including feed type, feed mill location, individual processing steps in the feed mill (such as before and after pelleting). These identified populations could also serve as indicator organism(s) for the likelihood of occurrence of *Salmonella*. This may be important if *Salmonella* occurs relatively infrequently in feeds and/or is dramatically reduced after antimicrobial treatments. Therefore, if based on natural contamination, screening of antimicrobials for control of *Salmonella* would be more difficult and identification of indicator organisms that are more frequent and parallel *Salmonella* behavior would have utility for routine testing.

## Formaldehyde, Feed Digestibility, and Potential Interaction with the Poultry GIT

Knowing the core feed microbial populations may be helpful not only for establishing effectiveness of antimicrobial treatments such as formaldehyde in the feed matrix but would also enhance understanding of the GIT microbial population response to formaldehyde treated feed as it enters the GIT. In most poultry studies, emphasis has been placed on antibacterial activities in either the feed matrix or the subsequent impact on *Salmonella* occurrence in the GIT of birds consuming treated feed. As more is becoming understood about the avian microbiome it is becoming possible to establish relationships between diets, dietary components and the specific responses of the avian microbial community. While this relationship has not been explored extensively with formaldehyde treated feeds there is indirect evidence of potential impact on the poultry GIT based on poultry performance and digestibility studies.

Wales et al. (4) concluded that formaldehyde, when applied as an antimicrobial feed additive, has not been generally shown as a cause of adverse responses in animals. However, Ricke (3) suggested given the dynamics of GIT digestion and microbial responses that a more detailed impact of formaldehyde on dietary protein availability for the concentrations of formaldehyde used as a feed antimicrobial treatment may also need to be considered. As more studies are conducted to examine the utility of formaldehyde as a chemical antimicrobial for poultry feed application, more specific nutritional responses such as amino acid and protein availability for digestion and absorption should also be taken into account in the overall determination of optimal concentrations to be used for antimicrobial applications.

Spears et al. (113) evaluated the impact of soybean meal treated with 0, 3, 6 or 9 kg/ton of formaldehyde (37%) on the performance of broiler chicks through 10 days of age. No adverse effects on body weight gain, feed consumption or feed conversion were observed at the 3 kg/ton treatment. At 6 kg/ton, feed intake was adversely affected. Spratt (57) reported no negative effect of high moisture corn diets treated with 2.5 kg/ton of formaldehyde (37%) in broilers (6 wks) or pullets (6 wks) or laying hens (20–33 wks). In more recent trials with broilers (114, 115), white layers (116), and brown layers (117), consumption of feed treated with a formaldehyde-based product (33% formaldehyde) at 2 to 3 kg/ton was not observed to negatively affect performance. The effect of higher levels of formaldehyde in feed has been evaluated in broilers and cockerels (118, 119). At 2.5 and 5 kg/ton of formaldehyde (37% solution), no adverse effects were reported. Feeding poultry 10 kg of formaldehyde/ton depressed feed intake, reduced body weight gain, and caused ulceration of the crop/gastrointestinal tract.

Barlow (27) was the first to examine the digestibility of formaldehyde in fishmeal destined for aquaculture. Fishmeal was treated with 0, 2, 4, or 6 kg/ton and fed to mink (test animal digestibility trials in aquaculture). No negative effects on protein digestibility occurred at levels up to 6 kg per ton. In digestibility trials in broilers, FBP treated feed/soybean meal was not observed to significantly affect protein digestibility broilers when fed at 2 kg/ton in both non-cecectomized broilers (115) and cecectomized broilers (120). Ironically, in both studies, protein digestibility was numerically improved but was not significantly different (*P* > 0.05). However, in both studies, it was not disclosed if the feed was subjected to pelleting thus the possibility of cross-linking of formaldehyde and amino acids at high reaction temperatures was not addressed. Jones et al. (121) conducted a study in which feed was treated with 3 kg/ton of a formaldehyde-based product and subjected to extreme pelleting conditions (86°C for 5.5 min). Feed was subsequently fed to cecectomized roosters (20 replicates/treatment), and amino acid digestibility determined. Formaldehyde was observed to not impact amino acid digestibility except for arginine (<1% reduction in digestibility).

While poultry performance and digestibility have been determined for birds fed formaldehyde treated feeds very little is known about the GIT microbial responses to these feeds. The lack of general influence on performance and digestibility would suggest that minimal impact occurs on the poultry GIT microbial populations except for the higher levels of formaldehyde when bird performance effects are noted. However, this does not rule out specific GIT microbial population responses in birds fed formaldehyde treated feeds. Historically, it was difficult to discern more subtle GIT microbial responses to differences in diets due to the limitations associated with culture methods for recovery of representative GIT microbial populations, particularly the more strict anaerobic GIT microorganisms. Development of molecular identification approaches such as NGS has made total GIT microbial populations much more accessible. As 16S rDNA sequencing methodologies for poultry microbiomes continue to advance it should be possible to achieve more indepth resolution for specific poultry GIT microbial population responses to formaldehyde treated feeds. Even when differences in overall poultry performance or digestibility responses are not detectable in the presence of formaldehyde treated feed, it is still possible that shifts in GIT could occur in response to changes in individual dietary components such as free amino acids and/or proteins. This response could vary depending on the particular GIT compartment in the bird such as the crop at the beginning of the GIT vs. the ceca at the terminal of the GIT. Not only are the microbial populations distinct in each of these GIT compartments but the lumen environment, pH, and metabolite composition are likely to be different as well (122–124). Application of metabolomics and transcriptomics may further reveal poultry GIT microbial responses even when detectable changes in GIT microbial populations composition do not occur.

## Conclusions

While a fairly wide range of chemical, physical and biological agents have been examined and in some cases commercially applied over the years as feed additives, formaldehyde remains one of the more frequently used from a commercial standpoint. It is considered effective as a feed additive, but it also may possess different antimicrobial mechanism(s) against *Salmonella* and other organisms such as GIT indigenous bacteria. However, its activity in the GIT, once consumed by the bird, may be different as well. It is conceivable since formaldehyde may bind directly to feed proteins that perhaps it is more stable in the GIT and therefore is more likely to reach the lower parts of the tract. It would be interesting to conduct studies on labeled formaldehyde similar to the work done by Hume et al. (125) with labeled propionate to determine whether that is indeed the case.

Formaldehyde can react with several different amino acids including the epsilon-amino group of lysine, the primary amide groups of asparagine and glutamine, the sulphydryl group of cysteine among others (23). This differential reactivity for particular amino acids could account for some of the variation seen in feed protein additive studies and the interaction with the GIT microbial community as proteins become modified with formaldehyde linkages and potentially present unique targets for protein hydrolysis by GIT microorganisms. In conclusion, the introduction of microbiome sequencing and bioinformatic tools should help to sort out some of the microbial ecology complexities associated with formaldehyde treated feeds both in the feed itself as well as once it is consumed by the bird.

## Author Contributions

All authors significantly contributed to the work of the current review. SR wrote the review with the assistance from DD and KR.

### Conflict of Interest Statement

KR is employed by the company Anitox Corporation, 1055 Progress Circle, Lawrence, GA 33043, USA. The remaining authors declare that the research was conducted in the absence of any commercial or financial relationships that could be construed as a potential conflict of interest.

## Footnotes

^1^Formaldehyde targets were based upon (58).
